# Comparison of side effects of oxytetracycline and talc pleurodesis: an experimental study

**DOI:** 10.1186/1749-8090-5-128

**Published:** 2010-12-13

**Authors:** Alper Gözübüyük, Berkant Özpolat, Ali Fuat Çiçek, Hasan Çaylak, Orhan Yücel, Kuthan Kavaklı, Sedat Gürkök, Onur Genç

**Affiliations:** 1 Department of Thoracic Surgery, GATA Military Medical Academy, Ankara, Turkey; 2 Department of Thoracic Surgery, Kırıkkale University, School of Medicine, Kırıkkale, Turkey; 3 Department of Pathology, GATA Military Medical Academy, Ankara, Turkey

## Abstract

**Background:**

Chemical pleurodesis is widely recommended in the treatment of refractory pleural effusion or pulmonary air leak of different etiologies. Although several agents have been used, many questions have remained unanswered about their toxicity. Talc is the most commonly used agent for the treatment, with rare, serious complications reported. Oxytetracycline pleurodesis in clinical practice has been described in a few studies, but literature reveals no experimental studies using this agent. We performed a prospective, randomized, observer-blinded, controlled study to evaluate the changes in lung histology and systemic response to pleurodesis with oxytetracycline and talc in acute and subacute phases in a rat model.

**Methods:**

Forty-two male albino Wistar rats were divided into three groups and 3 subgroups with 7 animals in each. Group 1 was given oxytetracycline, 35 mg/kg; Group 2 was given talc slurry, 60 mg/kg in 0.5 mL saline solution, and Group 3 was given only 0.5 mL saline intrapleurally. In subgroups "a" the nimls were sacrificed at the postoperative 72^nd ^hour and, in subgroups "b", on the postoperative day 7. The surfaces were graded by microscopic examination.

**Results:**

Oxytetracycline produced alveolar collapse, hemorrhage, edema, inflammation at the postoperative 72^nd ^hour and hemorrhage on the postoperative day 7, while talc produced significant edema, inflammation, proliferation, fibrosis at the postoperative 72^nd ^hour and hemorrhage, edema, inflammation, proliferation, and fibrosis on the postoperative day 7 (p < 0,0042). Talc produced significant edema compared to oxytetracycline on the postoperative day 7. On contralateral side, oxytetracycline and talc produced significant hemorrhage on the postoperative day 7 (p < 0.0042).

**Conclusions:**

Both agents were shown to produce pulmonary lesions. In acute phase, the pulmonary side effects of oxytetracycline were more pronounced, whereas the side effects of talc were prolonged to subacute phase. We propose that the occasional side effects in humans may be related to these changes as were observed in our rat model, and like talc, oxytetracycline must be used cautiously in patients with limited respiratory function.

## Background

Chemical pleurodesis is used to create fibrosis between pleural layers and obliterating pleural spaces to prevent fluid accumulation in malign diseases or benign diseases such as recurrent pleural effusion in cardiac failure, cirrhosis, nephritic syndrome, and chylothorax. It is also used in recurrent pneumothorax [1].

Talc is the most commonly tested and used agent for pleurodesis worldwide. Its use was first reported in 1935 by Bethune [2]. It is cheap, widely available, easy to use, and nearly 90% effective [3]. However, the success of this brilliant agent has been shadowed in clinical practice and clues from experimental studies that used this agent indicate potential risks for respiratory insufficiency, ARDS, and death [1,3-7].

Tetracycline has a wide range of efficacy (45-77%) as well. Main side effects of tetracycline, when used intrapleurally, are pain and fever. Tetracycline pleurodesis requires heavy analgesia, but serious pulmonary and extrapulmonary complications are not frequent [8]. Oxytetracycline pleurodesis is reported in a few studies in clinical applications but to the best of our knowledge, it has not been reported in any animal studies to date [9-11].

This prospective, randomized, observer-blinded, controlled study was conducted to evaluate the changes in pulmonary histology and systemic alterations after pleural administration of oxytetracycline and talc in acute and subacute phases in a rat experiment.

## Methods

Forty-two male albino Wistar rats (280-320 g, 6-8 months old) were provided by the research center of GATA MMA of the Faculty of Medicine. All animals received humane care in compliance with the European Convention on Animal Care and the study protocol was approved by the Animal Ethics Committee of GATA MMA (06/125). The animals were housed and operated the animal laboratory. Group 1 (n = 14) and Group 2 (n = 14) were the study groups, and Group 3 (n = 14) was the control group. The groups were further divided in two equal subgroups as "a" and "b" based on the time of sacrifice. In subgroups "a", the animals were sacrificed at the postoperative 72^nd ^hour and, in subgroups "b", the animals were sacrificed on the postoperative day 7 following the intrapleural administration of agents. In Group 1, the animals received intrapleural oxytetracycline (Pan-Terramycine, Pfizer, İstanbul. An injectable solution, which can be applied via IV, IM, SC and IP routes), 35 mg/kg; the animals in Group 2, 60 mg/kg of talc (the average particle size was 24.5 μ and fewer than 11% of the particles were smaller than 0.5 μ according to the producer) were given slurry in a total volume of 0.5 mL saline solution. Group 3 (the control group) received intrapleural 0.5 mL saline solution.

### Surgery

The rats were anesthetized with 35 mg/kg ketamine hydrochloride plus xylazine hydrochloride 5 mg/kg, administrated intramuscularly and under sterile conditions; a 5 mm skin incision was made over the seventh intercostal space. Oxytetracycline and talc slurry were introduced via a 16-gauge PTFE catheter into the left pleural space. The presence of air in the pleural space was checked, and if any, it was evacuated by using a three-way stopcock, and then the catheter was removed. The animals were rotated to assure distribution of agents to the entire pleural surface. The control group received intrapleural saline by the same method. The muscles and the skin were closed sequentially with 3/0 silk sutures. Animal movements were observed during the wake-up period for evidence of discomfort and pain (vocalization, tachypnea and restlessness), and when necessary, they received buprenorphine 1.3 mL, subcutaneously. The animals were maintained in adequate cages and fed according to the protocol of the animal quarters. There were no surgery related deaths or complications.

### Autopsy

Autopsies were performed by one of the investigators, who was blinded to the treatment received by the animals. In Groups 1a, 2a and 3a, the animals were sacrificed after 72 h, in groups 1b, 2b and 3b on day 7, under general anesthesia.

### Microscopy

Sections of the chest wall and both lungs were taken in the anteroposterior plane in the midlung zone including the mediastinal structures. The entire ipsilateral lung, en block chest wall with ipsilateral hemidiaphragm, as well as contralateral lung, heart with enblock mediastinal structures, chest wall, liver, and kidneys were collected: All were then placed in 10% buffered formalin, and the above mentioned samples were stained with hemotoxylin-eosin. Microscopic analysis was done by a pathologist blinded to the groups. The degree of microscopic lung disturbance characterized by alveolar collapse (*i.e.*, collapse of the framework involving alveolar sac and ducts leading to an overlap of the alveolar septa and reduction of the space for gas exchange), alveolar hemorrhage (*i.e.*, blood inside the alveolar spaces blurring the background structures), edema (*i.e.*, proteinaceous and amorphous material inside the alveolar space), cellular infiltrate (*i.e*., total number of cells in the alveoli) were evaluated as described by Vargas et al [3]. These parameters were subjectively semiquantified by a histopathologic score according to the extension and severity of the histopathologic lesions present in the lung tissue. The scoring was as follows: Grade 0, absent; grade 1, slight; grade 2, mild; grade 3, moderate; and grade 4, severe [3]. All the specimens in the talc group were submitted to polarized light with the purpose of investigating birefringent talc particles and were scored as 0; negative and 1, positive. The contralateral hemithorax was studied for the above mentioned changes. Macroscopical analysis was also done by the same pathologist blinded to the groups. As the sacrification time is short no dense adhesions were expected so the presence of adhesions was evaluated as no adhesions or minimal adhesions. Surrounding tissues (the diaphragm, liver, kidney, hearth, chest wall) were also examined.

### Statistical Analysis

Data analysis was performed by using SPSS for Windows, version 11.5 (SPSS Inc., Chicago, IL, United States). The data were shown as median (minimum-maximum). The differences among the groups were evaluated by Bonferroni Adjusted Kruskal-Wallis test. A *p *value less than 0.0125 was considered statistically significant. When the *p *value from Kruskal-Wallis test was statistically significant, Mann Whitney U multiple comparison test was used to determine the group that caused the difference. A *p *value less than 0.0042 was considered statistically significant. Whether the differences between days and lateralization were statistically significant or not were determined by Bonferroni Adjusted Mann Whitney U test.

## Results

Intrapleural administration of talc slurry did not cause distress in any of the animals, but after oxytetracycline instillation, 12 animals developed spasm, which indicated pain. These animals were supported with subcutaneously administered buprenorphine. All the subjects rapidly regained normal feeding and returned to normal activities.

### Macroscopy

When the pleural spaces were opened, there were only minimal adhesion at injection side between the pleural layers in 3 rats in talc injected group at the postoperative 72^nd ^hour. The lungs and other organs seemed normal. Visible talc deposits up to size of 1 mm were seen on the pleural surfaces in 8 subjects in the talc instilled groups. Except for the lungs, no talc particles were found in any of the visceral organs.

### Microscopy

**Figure 1:**
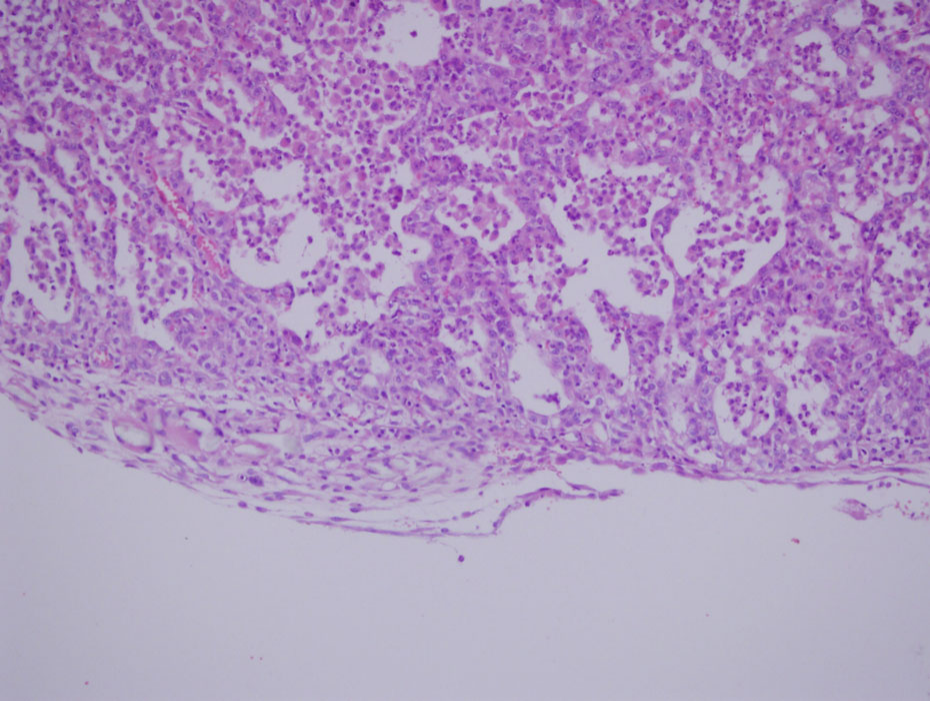
**Microscopic section of lung parenchyma exposed to oxytetracycline**. In the acute phase early signs of inflammation and alveolar collapse are seen. Hematoxylin and eosin, 200×.

**Figure 2:**
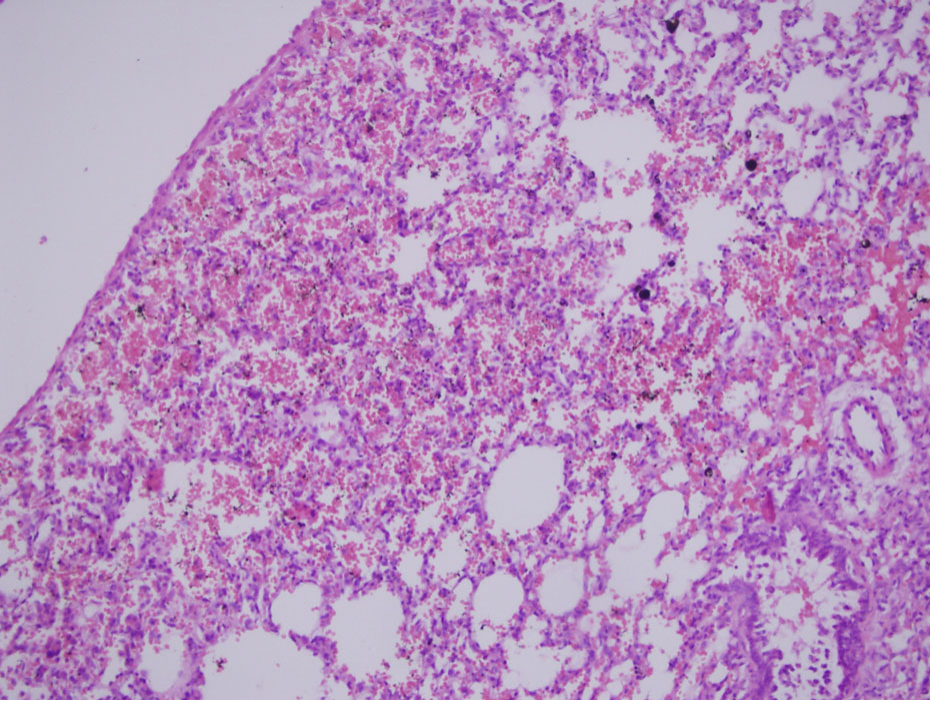
**Microscopic section of lung parenchyma exposed to oxytetracycline**. In the subacute phase signs of intraalveolar hemorrhage and alveolar collapse are seen. Hematoxylin and eosin, 200×.

**Figure 3:**
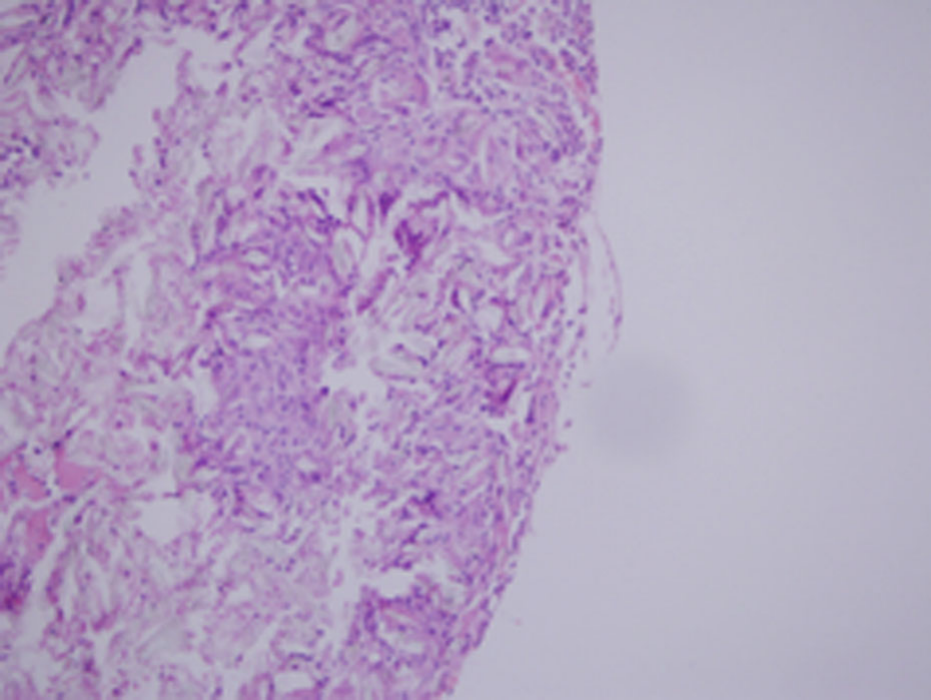
**Microscopic section of lung parenchyma exposed to talc at subacute phase**. Signs of inflammation and birefringent talc particles are seen. Hematoxylin and eosin, 200×.

Reactions to agents were in patchy manner in oxytetracycline groups, but diffuse and relatively more dense and severe in talc injected groups. The extension and distribution of the parenchymal changes were not homogeneous throughout the pulmonary tissue. Oxytetracycline produced significant alveolar collapse, hemorrhage, edema, inflammation compared to the control group at the postoperative 72^nd ^hour (p < 0.0042) (Figure 1). Oxytetracycline produced significant hemorrhage compared to the treatment in the control group on the postoperative day 7 (p < 0.0042) (Figure 2). Talc produced significant edema, inflammation, proliferation, fibrosis compared to the treatment in the control group at the postoperative 72^nd ^hour (p < 0.0042). Talc produced significant hemorrhage, edema, inflammation, proliferation, fibrosis compared to control group on the postoperative day 7 (p < 0.0042) (Figure 3). Talc produced significant edema compared to oxytetracycline on the postoperative day 7 (p < 0.0042). On the contralateral side, oxytetracycline and talc produced significant hemorrhage on the postoperative day 7 and talc produced significant edema both at the postoperative 72^nd ^hour and on the postoperative day 7 when compared to control group (p < 0.0042). The contralateral pleural surface, the liver, and the diaphragm of the animals did not show any inflammation. The scores of the microscopic exam of hematoxylin and eosin stained lung parenchyma of all the animals are shown in Table 1. No significant differences for birefringent talc particles were found in the talc group. Another important result of this study was although the pleural proliferation and fibrosis were significant in the talc group during acute and subacute phases, in the oxytetracycline group, no such changes were observed.

**Table 1:** The scores of microscopic examination of groups.

	Ipsilateral				Contralateral				Multiple Comparisons ^c^		
	
	OT	Talc	Control	***p***^**a**^	OT	Talc	Control	***p***^**b**^	***p***^**d**^	***p***^**e**^	***p***^**f**^
**AC**											
72 h	1 (0-1)	1 (1-2)^h^	0 (0-1)^h^	0.012	0 (0-1)	1 (0-1)	0 (0-1)	0.240	0.317	0.059	0.564
7 day	1 (0-1)	2 (0-4)	0 (0-1)	0.081	0 (0-1)	1 (0-4)	0 (0-1)	0.053	0.655	0.414	0.564
*p*^g^	1.000	0.535	1.000		0.710	0.259	1.000				

**Hem**											
72 h	1 (0-3)	2 (1-2)^h^	0 (0-0)^h^	0.002	0 (0-1)	1 (0-2)	0 (0-0)	0.067	0.119	0.038	1.000
7 day	1 (1-2)^i^	2 (1-4)^h^	0 (0-0)^h.i^	<0.001	1 (0-1)	2 (1-4)^h^	0 (0-0)^i^	<0.001	0.025	1.000	1.000
*p*^g^	0.805	0.805	1.000		0.383	0.017	1.000				

**Ede**											
72 h	1 (0-1)^i^	2 (1-2)^h^	0 (0-0)^h.i^	< 0.001	0 (0-1)	1 (1-1)^h^	0 (0-0)^h^	< 0.001	0.083	0.025	1.000
7 day	1 (0-1)^j^	2 (1-4)^h.j^	0 (0-0)^h^	< 0.001	1 (0-1)	1 (0-4)^h^	0 (0-0)^h^	0.005	0.564	0.025	1.000
*p*^g^	0.710	0.456	1.000		0.710	0.710	1.000				

**Inf**											
72 h	1 (1-2)^i^	2 (0-2)^h^	0 (0-0)^h.i^	0.002	0 (0-1)	1 (0-2)	0 (0-1)	0.200	0.023	0.129	0.317
7 day	1 (0-1)	2 (0-4)^h^	0 (0-0)^h^	0.003	0 (0-1)	1 (0-4)	0 (0-1)	0.014	0.157	0.336	0.317
*p*^g^	0.073	0.456	1.000		0.710	0.209	1.000				

**Prolif**											
72 h	0 (0-1)	1 (1-2)^h^	0 (0-0)^h^	< 0.001	0 (0-1)	1 (0-1)	0 (0-0)	0.070	1.000	0.014	1.000
7 day	1 (0-1)	1 (1-2)^h^	0 (0-0)^h^	< 0.001	0 (0-1)	1 (0-2)	0 (0-0)	0.062	0.564	0.157	1.000
*p*^g^	0.710	1.000	1.000		1.000	0.620	1.000				

**Fibr**											
72 h	1 (0-2)	1 (1-2)^h^	0 (0-0)^h^	0.003	0 (0-1)	0 (0-1)	0 (0-0)	0.128	0.102	0.025	1.000
7 day	0 (0-2)	1 (0-2)^h^	0 (0-0)^h^	0.011	0 (0-1)	0 (0-2)	0 (0-0)	0.138	0.655	0.083	1.000
*p*^g^	0.710	0.710	1.000		0.383	0.902	1.000				

**BTP**											
72 h	0 (0-0)	0 (0-1)	0 (0-0)	0.122	0 (0-0)	0 (0-1)	0 (0-0)	0.036	1.000	0.655	1.000
7 day	0 (0-0)	1 (0-1)	0 (0-0)	0.073	0 (0-0)	0 (0-0)	0 (0-0)	1.000	1.000	0.046	1.000
*p*^g^	1.000	0.383	1.000		1.000	0.209	1.000				

***Abbreviations; ****OT: oxytetracycline, AC: alveolar collapse, Hem: alveolar hemorrhage. Ede: edema, Inf: inflammation, Prolif: proliferation, Fibr: fibrosis, BTP: birefringent talc particles.*

Data were given as median (minimum-maximum),

a. comparisons of groups, ipsilateral side, 72 h and day 7 (Bonferroni Corrrection is applied and results were considered significant when p < 0.0125),

b. comparisons of groups, contralateral side, 72 h and day 7 (Bonferroni Corrrection is applied and results were considered significant when p < 0.0125),

c. comparisons of groups, ipsilateral-contralateral sides between 72 h and day 7 (Bonferroni Corrrection is applied and results were considered significant when p < 0.0083),

d. oxytetracycline group, comparisons of ipsilateral-contralateral sides,

e. talc group, comparisons of ipsilateral-contralateral sides,

f. control group, comparisons of ipsilateral-contralateral sides,

g. when groups compared, 72 h and day 7, (Bonferroni Corrrection is applied and results were considered significant when p < 0.0083),

h. the difference between Talc and control group is significant (p < 0.0042),

i. the difference between oxytetracycline and control group is significant (p < 0.0042),

j. the difference between oxytetracycline and talc group is significant (p < 0.0042).

## Discussion

Chemical pleurodesis is generally superior to mechanical pleurodesis in general practice because of its easy application without the need for general anesthesia, short hospital stay, and low cost. Many agents that are widely available, cheap, easy to use, effective, and/or safe have been defined for pleurodesis in literature [1,12]. However, none of these agents meets all these criteria [13]. Pleurodesis may affect neighboring or distant organs and tissues extrapleurally. Complications of pleurodesis were reported to be the most common with talc and tetracycline derivates [12,14].

The mechanism of transportation of chemical agents to the extra pleural organs is not well described, and the lymphatic way is one of them. The subpleural space of the visceral pleura and parietal pleura has a large network of lymphatic channels; the lymphatic drainage of the visceral pleura is primarily to the deep pulmonary plexus located in the interlobar and peribronchial spaces [15]. It was postulated that absorbed materials moves into the lymphatic system and are transported to the mediastinal lymph nodes and thoracic duct and finally to systemic circulation [5]. Another hypothesis is the acute pneumonitis, which is related to the systemic absorption of especially smaller talc particles and the subsequent inflammatory reactions in the lungs [7]. It has been demonstrated in animal models that systemic absorption of talc causes distant embolisation to the lungs, liver, spleen, brain, kidney, heart, skeletal muscle, and even the brain [6,7]. It has been shown in many experiments that pleura as a barrier between pleural spaces and lung parenchyma is destroyed after the instillation of agents used for pleurodesis, which leads to transpleural diffusion. By this way, the agent may easily penetrate into the lung parenchyma and cause unwanted side effects [6].

In the light of these facts, this rat model was developed to determine whether oxytetracycline may cause lung damage like talc in acute and subacute phases of chemical pleurodesis.

Our results support that both agents must be used cautiously and should be avoided in patients with limited pulmonary reserve. These changes were observed with both oxytetracycline and talc administrations and in the acute and subacute phases due to systemic distribution. Moreover, morphologic changes were observed on the contralateral side.

Well-documented side effects of talc pleurodesis are fever (16-69%) and chest pain (7%). After intrapleural administration as slurry or insufflation, serious pulmonary complications, including acute pneumonitis, acute respiratory failure and ARDS with different incidences ranging between 0% and 33% have been reported [7], and in some cases, this complication was lethal [6]. Previous experimental studies demonstrated pleural and pulmonary acute inflammatory responses to talc pleurodesis, which were pleural thickening, fibrin deposition in areas of mesothelial denudement and transient mononuclear vasculitis noted in rabbit lung [6,16]. Montes et al also found focal inflammatory responses around of talc particles, capillary vasodilation and hyperemia in pulmonary parenchyma and foreign body granulomas as a consequence of pleural talc depositions [17]. However, in some studies, no significant histological alteration was found within the subjacent lung parenchyma [18,19]. In our study, talc produced significant alveolar edema and inflammation in the acute phase and in the subacute phase, these changes were added by alveolar hemorrhage. In this study, the inflammation of contralateral lungs was also shown in detail. On the contralateral side, it produced significant edema in the acute phase and hemorrhage and edema in the subacute phase. Considering the high rate of contralateral changes reported after talc poudrage we could at least theoretically justified the occurence of ARDS. In a rabbit study, birefringent talc bodies were found in abdominal organs in 15-40% of the animals studied, and another study showed that all the extrathoracic organs contained birefringent talc particles [4,16]. In our study, no significant differences were found in the talc group when compared to the control group for birefringent talc particles.

For tetracycline pleurodesis, the most commonly reported adverse effects were pain and fever. Tetracycline requires sedation with benzodiazepines or analgesia with a narcotic drug. In addition, vestibular symptoms and after high doses, hemothorax were observed in animal studies [14,20]. After tetracycline pleurodesis, systemic absorption led to acute renal failure and hepatotoxicty in animal models, and similar to talc, the administration of a tetracycline derivative doxycycline was reported to lead to the development of the acute respiratory distress syndrome and even death [21]. Wooten et al showed that tetracycline was systemically absorbed following intrapleural instillation. They explained the entrance of these agents beyond the extrapleural space and systemic circulation by/with lymphatic absorption from pleural surfaces and by exposing the subpleural microvessels and microlymphatics of the loose connective tissue layer directly to the contents of the pleural space by denudement of mesothelial cells after administration of sclerosing agents [22]. After the discontinuation of production of the injectable tetracycline hydrochloride by the manufacturer, alternative forms like doxycycline and minocycline have been used for pleurodesis [1,13,14].

Oxytetracycline, a derivative of Streptomyces rimosus, was introduced in 1950 and has been an easily accessible drug in Turkey [10]. However, it was published in few clinical studies probably due to some ethical reasons [9-11]. Furthermore, there are no experimental studies on oxytetracycline pleurodesis in the literature. Şenyigit et al administered oxytetracycline at a dose of 35 mg/kg, and it was well tolerated by patients with minor side effects like nausea-vomiting and hypotension (4.3%), chest pain (30.4%), fever (23.1%). Thus, the authors considered the results acceptable [10]. Yıldırım et al have reported only pleuritic pain which was managed by intrapleurally analgesia [9]. The reports relating to systemic changes after oxytetracycline pleurodesis in clinical studies are not sufficient to draw a conclusion, but side effects were reported to be minor and acceptable. In our study, we observed signs of pain in 12 rats, which was relieved with analgesics. We found that oxytetracycline produced significant alveolar collapse, hemorrhage, edema, inflammation in the acute phase and only hemorrhage in the subacute phase. On the contralateral side, oxytetracycline produced significant hemorrhage in the subacute phase. These findings show that pulmonary toxicity of oxytetracycline decreases rapidly when compared to talc; in our study, the effects on the contralateral side were similar. So this data may confirm the difference of mechanisms creating inflammatory response for each agent.

In this study, talc produced pleural proliferation and fibrosis starting from the acute phase of administration. Nevertheless, with oxytetracycline, no such findings were found. We are currently developing an animal model for oxytetracycline pleurodesis to further evaluate the effect of different concentrations for successful pleurodesis in a long period.

The major limitation of this study for clinical translation is the lack of drainage of oxytetracycline after infusion into the pleural space.

## Conclusions

The results of this study showed alterations in the lung anatomy after pleurodesis procedure with oxytetracycline and talc. The alterations occured bilaterally in the lungs and constitute clues for possible fatal outcomes with both agents. Our results suggest that in clinical translation chemical pleurodesis can easily create mortal outcomes in patients with limited pulmonary functions.

## Competing interests

The authors declare that they have no competing interests.

## Authors' contributions

AG and BÖ conceived of the study, and participated in its design and coordination and helped to draft and performed the statistical analysis. AFÇ carried out the macroscopic and microscopic studies. HÇ, OY and KK participated in the design of the study. SG and OG participated in the sequence alignment and drafted the manuscript. All authors read and approved the final manuscript.
